# Revised diagnostic criteria for neurofibromatosis type 1 and Legius syndrome: an international consensus recommendation

**DOI:** 10.1038/s41436-021-01170-5

**Published:** 2021-05-19

**Authors:** Eric Legius, Ludwine Messiaen, Pierre Wolkenstein, Patrice Pancza, Robert A. Avery, Yemima Berman, Jaishri Blakeley, Dusica Babovic-Vuksanovic, Karin Soares Cunha, Rosalie Ferner, Michael J. Fisher, Jan M. Friedman, David H. Gutmann, Hildegard Kehrer-Sawatzki, Bruce R. Korf, Victor-Felix Mautner, Sirkku Peltonen, Katherine A. Rauen, Vincent Riccardi, Elizabeth Schorry, Anat Stemmer-Rachamimov, David A. Stevenson, Gianluca Tadini, Nicole J. Ullrich, David Viskochil, Katharina Wimmer, Kaleb Yohay, International Consensus Group on Neurofibromatosis Diagnostic Criteria (I-NF-DC), Alicia Gomes, Justin T. Jordan, Victor Mautner, Vanessa L. Merker, Miriam J. Smith, David Stevenson, Monique Anten, Arthur Aylsworth, Diana Baralle, Sebastien Barbarot, Fred Barker, Shay Ben-Shachar, Amanda Bergner, Didier Bessis, Ignacio Blanco, Catherine Cassiman, Patricia Ciavarelli, Maurizio Clementi, Thierry Frébourg, Marco Giovannini, Dorothy Halliday, Chris Hammond, C. O. Hanemann, Helen Hanson, Arvid Heiberg, Pascal Joly, Michel Kalamarides, Matthias Karajannis, Daniela Kroshinsky, Margarita Larralde, Conxi Lázaro, Lu Le, Michael Link, Robert Listernick, Mia MacCollin, Conor Mallucci, Christopher Moertel, Amy Mueller, Joanne Ngeow, Rianne Oostenbrink, Roger Packer, Laura Papi, Allyson Parry, Juha Peltonen, Dominique Pichard, Bruce Poppe, Nilton Rezende, Luiz Oswaldo Rodrigues, Tena Rosser, Martino Ruggieri, Eduard Serra, Verena Steinke-Lange, Stavros Michael Stivaros, Amy Taylor, Jaan Toelen, James Tonsgard, Eva Trevisson, Meena Upadhyaya, Ali Varan, Meredith Wilson, Hao Wu, Gelareh Zadeh, Susan M. Huson, D. Gareth Evans, Scott R. Plotkin

**Affiliations:** 1 Department of Human Genetics, KU Leuven and University Hospital, Leuven, Belgium; 2 Department of Genetics, University of Alabama at Birmingham, Birmingham, AL USA; 3 Assistance Publique-Hôpital Paris (AP-HP), Hôpital Henri-Mondor, UPEC, Service de Dermatologie, Créteil, France; 4 Children’s Tumor Foundation, New York, NY USA; 5 Division of Ophthalmology, The Children’s Hospital of Philadelphia, Philadelphia, PA USA; 6 Clinical Genetics, Royal North Shore Hospital, St. Leonards, NSW, Australia and University of Sydney, Sydney, Australia; 7 Johns Hopkins Comprehensive Neurofibromatosis Center, Baltimore, MD USA; 8 Department of Clinical Genomics, Mayo Clinic College of Medicine, Rochester, MN USA; 9 Department of Pathology, Universidade Federal Fluminense, Niteroi, Brasil; 10 Neurology, Guy’s and St. Thomas’ Hospital and NHS Trust, London, UK; 11 Division of Oncology, The Children’s Hospital of Philadelphia, Philadelphia, PA USA; 12 Medical Genetics, University of British Columbia, Vancouver, BC Canada; 13 Neurology, Washington University, St. Louis, MO USA; 14 Institute of Human Genetics, University of Ulm, Ulm, Germany; 15 Neurology, University Hospital of Hamburg-Eppendorf, Hamburg, Germany; 16 Dermatology, University of Turku and Turku University Hospital, Turku, Finland; 17 Department of Dermatology and Venereology, University of Gothenburg, Gothenburg, Sweden; 18 Pediatrics, University of California Davis, Sacramento, CA USA; 19 The Neurofibromatosis Institute, La Crescenta, CA USA; 20 Medical Genetics, Cincinnati Children’s Hospital Medical Center, Cincinnati, OH, USA; 21 Neuropathology, Massachusetts General Hospital, Boston, MA USA; 22 Medical Genetics, Stanford University, Stanford, CA USA; 23 Pediatric Dermatology, University of Milan, Milan, Italy; 24 Department of Neurology, Boston Children’s Hospital, Boston, MA USA; 25 Medical Genetics, University of Utah, Salt Lake City, UT USA; 26 Institute of Human Genetics, Medical University of Innsbruck, Innsbruck, Austria; 27 NYU Langone Health, New York, NY USA; 28 Clinical Genetics, (Formerly) Manchester Center for Genomic Medicine, Manchester University Hospitals, NHS Foundation Trust, Manchester, UK; 29 Department of Genomic Medicine, St Mary’s Hospital, Manchester Academic Health Sciences Centre (MAHSC), Division of Evolution and Genomic Science, University of Manchester, Manchester, UK; 30 Department of Neurology and Cancer Center, Massachusetts General Hospital, Boston, MA USA; 31 Department of Neurology, Maastricht University Medical Center, Maastricht, Netherlands; 32 Department of Genetics, University of North Carolina, Chapel Hill, NC USA; 33 Department of Human Development and Health, University of Southampton, Southampton, UK; 34 Centre Hospitalier Universitaire de Nantes, Nantes, France; 35 Department of Neurosurgery, Massachusetts General Hospital, Boston, MA USA; 36 Clalit Research Institute, & Schneider Children’s Medical Center, Ramat-Gan, Israel; 37 Department of Genetics and Development, Columbia University Medical Center, New York, NY USA; 38 Department of Dermatology, Centre Hospitalier Universitaire de Montpellier, Montpellier, France; 39 Department of Clinical Genetics, Hospital Universitari Germans Trias I Pujol, Badalona, Spain; 40 Department of Ophthalmology, University Hospitals Leuven, Leuven, Belgium; 41 Department of Neurosurgery, Hospital de Clinicas Gral San Martin, San Martin, Argentina; 42 Department of Clinical Genetics, University of Padova, Padova, Italy; 43 Department of Genetics, University Hospital Rouen, Rouen, France; 44 Department of Head and Neck Surgery, David Geffen School of Medicine, University of California–Los Angeles, Los Angeles, CA USA; 45 Department of Clinical Genetics, Oxford University Hospitals NHS Foundation Trust, Oxford, UK; 46 Department of Ophthalmology, King’s College London, London, UK; 47 Institute of Translational and Stratified Medicine, Peninsula Medical School, University of Plymouth, Plymouth, UK; 48 Department of Genetics, St George’s University Hospitals, London, UK; 49 Department of Medical Genetics, Oslo University Hospital, Oslo, Norway; 50 Department of Dermatology, University Hospital Rouen, Rouen, France; 51 Department of Neurosurgery, Hôpital Pitié Salpêtrière, Paris, France; 52 Department of Pediatrics and Otolaryngology, Memorial Sloan Kettering Cancer Center, New York, NY USA; 53 Department of Dermatology, Massachusetts General Hospital, Boston, MA USA; 54 Department of Dermatology, Hospital Aleman, Buenos Aires, Argentina; 55 Institut Català d’Oncologia (ICO-IDIBELL-CIBERONC), Hospitalet de Llobregat, Barcelona, Spain; 56 Department of Dermatology, University of Texas, Southwestern, Dallas, TX USA; 57 Department of Neurosurgery and Otolaryngology, Mayo Clinic, Rochester, MN USA; 58 Department of Pediatrics, Ann and Robert H Lurie Children’s Hospital of Chicago, Chicago, IL USA; 59 Bend, OR USA; 60 Department of Neurosurgery, Alder Hey Children’s Hospital NHS, Liverpool, UK; 61 Department of Pediatrics, University of Minnesota, Minneapolis, MN USA; 62 Cancer Genetics, Massachusetts General Hospital, Boston, MA USA; 63 Lee Kong Chian School of medicine, Nanyang Technological University, Singapore and Cancer Genetics Service, National Cancer Center, Singapore, Singapore; 64 Department of Pediatrics, Erasmus University Medical Center, Rotterdam, Netherlands; 65 The Brain Tumor Institute, Gilbert Family Neurofibromatosis Institute, Children’s National Medical Center, Washington, DC USA; 66 Department of Experimental and Clinical Biomedical Science “Mario Serio”, University of Florence, Florence, Italy; 67 Department of Neurology, John Radcliff Hospital, Oxford, UK; 68 Institute of Biomedicine, University of Turku and Turku University Hospital, Turku, Finland; 69 National Institutes of Health/National Institute of Arthritis and Musculoskeletal and Skin Diseases, Bethesda, MD USA; 70 Department of Medical Genetics, University Hospital Ghent, Ghent, Belgium; 71 School of Medicine, Federal University of Minas Gerais, Belo Horizonte, Brazil; 72 Department of Neurology, Children’s Hospital Los Angeles, Los Angeles, CA USA; 73 Department of Clinical and Experimental Medicine, Section of Pediatrics and Child Neuropsychiatry, University of Catania, Catania, Italy; 74 The Institute for Health Science Research Germans Trias i Pujol (IGTP), Barcelona, Spain; 75 Center of Medical Genetics, Munich, Germany; 76 Division of Informatics, Imaging and Data Sciences, University of Manchester, Manchester, UK; 77 Clinical Genetics Department, Cambridge University Hospitals NHS Foundation Trust, Cambridge, UK; 78 Department of Pediatrics, University Hospital Leuven, Leuven, Belgium; 79 Department of Pediatrics and Neurology, University of Chicago Medicine, Chicago, IL USA; 80 Institute of Cancer Genetics, Cardiff University, Cardiff, UK; 81 Department of Pediatric Oncology, Hacettepe University, Ankara, Turkey; 82 Department of Clinical Genetics, Children’s Hospital Westmead, Sydney, Australia; 83 Shanghai Ninth People’s Hospital Affiliated Shanghai Jiao Tong University School of Medicine, Shangai, China; 84 Princess Margaret Cancer Centre, Toronto Western Hospital, Toronto, ON Canada

## Abstract

**Purpose:**

By incorporating major developments in genetics, ophthalmology, dermatology, and neuroimaging, to revise the diagnostic criteria for neurofibromatosis type 1 (NF1) and to establish diagnostic criteria for Legius syndrome (LGSS).

**Methods:**

We used a multistep process, beginning with a Delphi method involving global experts and subsequently involving non-NF experts, patients, and foundations/patient advocacy groups.

**Results:**

We reached consensus on the minimal clinical and genetic criteria for diagnosing and differentiating NF1 and LGSS, which have phenotypic overlap in young patients with pigmentary findings. Criteria for the mosaic forms of these conditions are also recommended.

**Conclusion:**

The revised criteria for NF1 incorporate new clinical features and genetic testing, whereas the criteria for LGSS were created to differentiate the two conditions. It is likely that continued refinement of these new criteria will be necessary as investigators (1) study the diagnostic properties of the revised criteria, (2) reconsider criteria not included in this process, and (3) identify new clinical and other features of these conditions. For this reason, we propose an initiative to update periodically the diagnostic criteria for NF1 and LGSS.

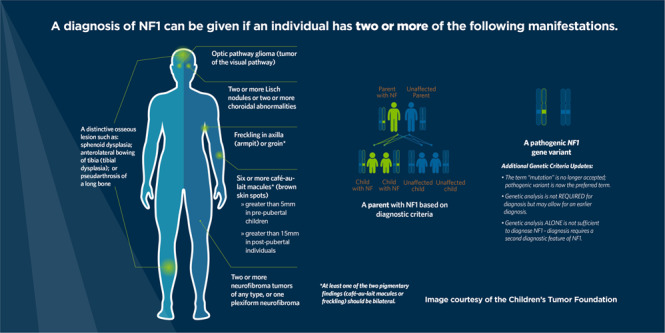

## INTRODUCTION

### Nomenclature and history

Neurofibromatosis type 1 (NF1; OMIM 613113), inherited in an autosomal dominant pattern, is characterized by multiple café-au-lait macules (CALMs), skinfold freckling (more correctly termed lentiginous macules since they occur in non–sun exposed areas), iris Lisch nodules, tumors of the nervous system, and other features.^1,2^ Disease manifestations can occur in any body system. There is a significantly increased risk of certain cancers, including female breast cancer <50 years, malignant peripheral nerve sheath tumors (MPNSTs), and brain tumors.^3^ The modern history of nomenclature of neurofibromatosis started in 1987 with the National Institutes of Health (NIH) Consensus Development Conference on Neurofibromatosis.^4^ Until that time there had been confusion in the literature as to whether bilateral vestibular schwannomas (previously termed acoustic neuromas) were a feature of NF1 or a distinct entity. Riccardi laid the groundwork for the NIH consensus criteria by proposing a numerical classification system based on the presence/absence of CALMs and skinfold freckling, specific eye signs, neurofibromas, and complications specific to each type.^5^ The mapping of the *NF1* gene to chromosome 17^6^ and the *NF2* gene to chromosome 22^7^ allowed the consensus conference to establish diagnostic criteria for the two conditions (Table S1). The 1988 panel recognized that “[p]atients who do not fit into the NF1 or NF2 group by clinical or genetic criteria may, in the future, constitute the basis for establishing additional types of NF.”^4^

The NIH criteria include the most frequent disease manifestations (CALMs, freckling, neurofibromas, and Lisch nodules) alongside disease complications typical of NF1. Since 1987 there has been one formal review of the NF1 criteria by the Clinical Care Advisory Board of the National Neurofibromatosis Foundation (now the Children’s Tumor Foundation).^8^ No criteria alterations were suggested.

The *NF1* gene was cloned in 1990.^9,10^ Subsequent cell biology studies have found that neurofibromin, the *NF1* gene product, largely operates as a GTPase-activating protein (GAP) that negatively regulates the RAS/MAPK pathway activity by accelerating the hydrolysis of RAS-bound GTP.^1^ Cell biology advances and animal models have led to the identification of MEK inhibitors as a treatment for plexiform neurofibromas.^11^ In April 2020, selumetinib was approved by the US Food and Drug Administration for treatment of children with NF1-related symptomatic plexiform neurofibroma.^11^

NF1 was the first human condition mapped to the RAS pathway but subsequently other conditions have been shown to be caused by pathogenic variants (PVs) in RAS pathway genes, including Noonan, Costello, and cardiofaciocutaneous syndromes.^12^ Together, this group of conditions is referred to as RASopathies. Legius syndrome (LGSS; OMIM 611431) is the RASopathy with the most overlap to NF1.^13^ LGSS, inherited in an autosomal dominant pattern, is characterized by multiple CALMs similar to NF1 with or without skinfold freckling, caused by heterozygous PVs in *SPRED1*, located on chromosome 15.^13,14^ Further studies have shown that *SPRED1* PVs account for 1% of sporadic cases and 19% of familial cases with pigmentary features of NF1 (CALMs and freckling) only.^14^ No individuals with LGSS have developed Lisch nodules, neurofibromas, or other complications of NF1. LGSS could be clinically diagnosed as NF1 using the NIH criteria (50% satisfy the criteria^14^) but it has a different natural history. The condition is less frequent than NF1 with an estimated birth prevalence of 1/46,000–1/75,000 (4% of individuals followed at a neurofibromatosis clinic),^15^ compared to 1/2,000–1/3,000 for NF1.^16^

The identification of LGSS highlights an important limitation of the NIH NF1 clinical diagnostic criteria. In addition, other conditions with overlapping phenotypes but distinct natural histories have been identified or better defined (e.g., segmental/mosaic NF1^17^ and constitutional mismatch repair deficiency [CMMRD] syndrome^18^) and new potential criteria for NF1 have been identified (e.g., choroidal anomalies^19^ and nevus anemicus^20^). Finally, *NF1* genetic testing has become clinically available with a high detection rate^21^ and clinically useful genotype–phenotype correlations have been identified.^22^ With sponsorship from the Children’s Tumor Foundation (CTF), an international panel of neurofibromatosis and schwannomatosis experts was assembled in 2017 and charged with reviewing diagnostic criteria for NF1, NF2, and schwannomatosis. The work on NF2 and schwannomatosis is presented in another paper.

## MATERIALS AND METHODS

A modified Delphi process was used to reach consensus on revised diagnostic criteria (Supplementary Fig. 1). A steering committee (seven experts) reviewed the literature and generated the statements for the two rounds of the Delphi process (data S1, S2, S3, S4, S5, and Supplemental Material and Methods) regarding potential changes to NF1 diagnostic criteria. The Steering Committee actively sought input from patients, families, and advocates regarding the proposed criteria and the new diagnostic criteria were finalized in January 2020. All clinical images are used with consent from the patient/patient’s parent.

## RESULTS

### Development of initial proposals to revision of NF1 diagnostic criteria

The steering committee evaluated the literature for clinical and/or genetic features that could reliably identify and distinguish NF1, mosaic NF1, LGSS, and mosaic LGSS. Among the clinical features considered were presence of characteristic tumors such as neurofibromas or gliomas; presence of cutaneous findings including CALMs, skinfold freckling, juvenile xanthogranulomas,^23^ and nevus anemicus;^20^ presence of characteristic ophthalmologic findings such as Lisch nodules and choroidal abnormalities;^24^ presence of characteristic osseous lesions such as sphenoid wing dysplasia, scoliosis, anterolateral bowing of the lower leg, pseudarthrosis;^25^ presence of focal areas of signal intensity (FASI) detected by brain magnetic resonance image (MRI),^26^ and presence of a family history. Among the molecular features considered were identification of an *NF1* and *SPRED1* pathogenic variant (PV) in unaffected tissue.

### Modified Delphi process

Sixty-seven of 76 NF experts (89%) participated in the first Delphi process by rating the 13 NF1 diagnostic criteria statements (Supplementary Fig. 2). There was very high consensus (median score = 10/10) for four proposed changes: revising criteria to clearly define mosaic NF1; adding genetic diagnosis, without implying that genetic testing is required or recommended for diagnosis; replacing thinning of a long bone by anterolateral bowing of the lower limb; and retaining the specified criteria regarding neurofibromas, optic glioma, and Lisch nodules with minor wording changes for the latter two criteria. There was high consensus (median score = 7–9/10) for seven proposed changes: revising the nomenclature to clearly distinguish NF1/LGSS from NF2/schwannomatosis; clarifying which first-degree relatives meet criteria for family history; combining CALMs and skinfold freckling into a single criterion; naming individual long bones commonly affected by congenital bowing and/or pseudarthrosis; and adding juvenile xanthogranulomas, choroidal abnormalities, and dystrophic scoliosis to the revised criteria. There was no consensus (median score = 4–6/10) for two proposed changes: adding nevus anemicus and FASI to the revised criteria.

At the 2018 meeting in New York, working groups developed nine revised statements concerning diagnostic criteria for discussion, including discussion about changing the format of the diagnostic criteria into a tiered system with A and B features. Fifty-eight of 76 NF experts (76%) participated by rating the nine revised statements (Supplementary Fig. 3). There was very high consensus (more than 80% agreement) for four proposed changes: adding a new criterion of genetic diagnosis, and recommending genetic testing for patients with segmental clinical findings, for families with two affected siblings and clinically unaffected parents, and for children who meet the criteria based on pigmentary findings alone. There was high consensus (agreement 60–80%) for three proposed changes: considering genetic testing for individuals with multiple bilateral spinal nerve neurofibromas without other features of NF1, requiring use of slit lamp or indirect ophthalmoscopy for identification of Lisch nodules, and including choroidal abnormalities as a diagnostic criterion. Finally, there was more agreement to retain the current format as opposed to selecting A/B criteria (71% agreement). In total, 74/76 NF experts (97%) were involved in the revision process (data S4).

Nine of 12 (75%) non-NF specialists responded to the survey (data S5) and strongly agreed with the proposed changes to the NF1 diagnostic criteria. The experts agreed that nonspecialists would be able to use the revised criteria.

### Proposed new diagnostic criteria for NF1 and LGSS

Ultimately, consensus was reached on the minimal clinical and genetic criteria for diagnosing NF1 and LGSS. Final recommendations for NF1 are listed in Table 1 and for LGSS in Table 2.

**Table 1:** Revised diagnostic criteria for neurofibromatosis type 1 (NF1).

A: The diagnostic criteria for NF1 are met in an individual who does not have a parent diagnosed with NF1 if two or more of the following are present:
• Six or more café-au-lait macules over 5 mm in greatest diameter in prepubertal individuals and over 15 mm in greatest diameter in postpubertal individuals^a^ (Supplementary Fig. 6)
• Freckling in the axillary or inguinal region^a^ (Supplementary Fig. 7)
• Two or more neurofibromas of any type *or* one plexiform neurofibroma (Supplementary Fig. 8a, b)
• Optic pathway glioma (Supplementary Fig. 9)
• Two or more iris Lisch nodules identified by slit lamp examination or two or more choroidal abnormalities (CAs)—defined as bright, patchy nodules imaged by optical coherence tomography (OCT)/near-infrared reflectance (NIR) imaging (Supplementary Fig. 10a, b)
• A distinctive osseous lesion such as sphenoid dysplasia,^b^ anterolateral bowing of the tibia, or pseudarthrosis of a long bone (Supplementary Fig. 11)
• A heterozygous pathogenic *NF1* variant with a variant allele fraction of 50% in apparently normal tissue such as white blood cells
B: A child of a parent who meets the diagnostic criteria specified in A merits a diagnosis of NF1 if one or more of the criteria in A are present

^a^If only café-au-lait macules and freckling are present, the diagnosis is most likely NF1 but exceptionally the person might have another diagnosis such as Legius syndrome. At least one of the two pigmentary findings (café-au-lait macules or freckling) should be bilateral.

^b^Sphenoid wing dysplasia is not a separate criterion in case of an ipsilateral orbital plexiform neurofibroma.

**Table 2:** Diagnostic criteria for Legius syndrome.

A: The diagnostic criteria for Legius syndrome are met in an individual who does not have a parent diagnosed with Legius syndrome if the following CRITERIA are present:
• Six or more café-au-lait macules (Supplementary Fig. 12) bilaterally distributed and no other NF1-related diagnostic criteria except for axillary or inguinal freckling^a^
• A heterozygous pathogenic variant in *SPRED1* with a variant allele fraction of 50% in apparently normal tissue such as white blood cells
B: A child of a parent who meets the diagnostic criteria specified in A merits a diagnosis of Legius syndrome if one or more of the criteria in A are present

^a^The presence of fewer than six café-au-lait spots does not exclude Legius syndrome.

### Proposed diagnostic criteria for mosaic NF1 and mosaic LGSS

The final recommendations for diagnostic criteria for mosaic NF1 are listed in Table 3, and for mosaic LGSS in Table 4. The criteria allow for both a molecular and clinical diagnosis of mosaic NF1 and mosaic LGSS, although mosaic LGSS is very rare.^27^

**Table 3:** Diagnostic criteria for mosaic neurofibromatosis type 1 (NF1).

The diagnostic criteria for mosaic NF1 are met in an individual if any of the following is present:
1. A pathogenic heterozygous *NF1* variant with a variant allele fraction of significantly less than 50% in apparently normal tissue such as white blood cells AND one other NF1 diagnostic criterion (except a parent fulfilling diagnostic criteria for NF1)
2. An identical pathogenic heterozygous *NF1* variant in two anatomically independent affected tissues (in the absence of a pathogenic *NF1* variant in unaffected tissue)^a^
3. A clearly segmental distribution of café-au-lait macules or cutaneous neurofibromas AND a. Another NF1 diagnostic criterion (except a parent fulfilling diagnostic criteria for NF1)^b^ or b. Child fulfilling diagnostic criteria for NF1
4. Only one NF1 diagnostic criterion from the following list: freckling in the axillary and inguinal region, optic pathway glioma, two or more Lisch nodules or two or more choroidal abnormalities, distinctive osseous lesion typical for NF1, two or more neurofibromas or one plexiform neurofibroma AND a child fulfilling the criteria for NF1

^a^Neurofibroma and overlying hyperpigmented skin count for one tissue only; different tissues originating from the same primary affected lesion count for one tissue only.

^b^If only café-au-lait macules and freckling are present, the diagnosis is most likely mosaic neurofibromatosis type 1 but rarely might be mosaic Legius syndrome or constitutional mismatch repair deficiency (CMMRD) syndrome.

**Table 4:** Diagnostic criteria for mosaic Legius syndrome.

The diagnostic criteria for mosaic Legius syndrome are met in an individual if any of the following is present:
1. A heterozygous pathogenic *SPRED1* variant with a variant allele fraction of significantly less than 50% in apparently normal tissue such as white blood cells AND six or more café-au-lait macules
2. An identical pathogenic heterozygous *SPRED1* variant in two independent affected tissues (in the absence of a pathogenic *SPRED1* variant in unaffected tissue)^a^
3. A clearly segmental distribution of café-au-lait macules AND a child fulfilling the criteria for Legius syndrome

^a^Different tissues originating from the same primary affected lesion count for one tissue only.

## DISCUSSION

In this paper, the results of an international, multispecialty effort to revise the diagnostic criteria for NF1 and LGSS are presented. The process extended over three years, reflecting the commitment to involve a wide array of specialists, nonspecialists, patient advocacy groups, patients, and family members into the process. The goal was to incorporate clinical and genetic discoveries made since the initial consensus conference into the revised criteria. We used a modified Delphi approach to reach maximum consensus among stakeholders with the understanding that complete agreement was not a reasonable goal. A recurring theme during the process was the challenge of balancing the expectations of different medical specialties for diagnostic criteria. For example, some specialists emphasized the importance of diagnosing children at a young age to screen for important medical features (i.e., high sensitivity) while others emphasized the need to avoid potential misdiagnosis (i.e., high specificity). Ultimately, the group attempted to choose criteria that balanced the needs of high sensitivity and specificity. The Delphi process worked well in both reaching consensus in some areas and highlighting the issues with varying opinion for discussion at the meeting in New York.

### Pigmentary findings alone are not specific for NF1

The current clinical diagnostic criteria have low sensitivity in children since diagnostic signs appear progressively over time.^1^ Participants considered, but did not ultimately recommend, combining pigmentary criteria (CALMs/skinfold freckling) into a single criterion. Although combining would mean patients with LGSS would not meet the criteria for NF1, it could potentially delay diagnosis in children without access to molecular testing. To alert clinicians to the differential diagnosis, an asterisk was added to the diagnostic criteria for NF1 stating that if only pigmentary findings are present, clinicians should consider alternative diagnoses, including, but not limited to, LGSS, Noonan syndrome with multiple lentigines, and CMMRD.

The challenge of distinguishing NF1 from LGSS based on pigmentary findings was demonstrated by Messiaen et al.^14^ who studied an anonymous cohort of 2,432 individuals referred for *NF1* molecular diagnosis. An *NF1* PV was identified in 1,114 individuals and a *SPRED1* PV in 33 (2.9% of PV-positive individuals). In the subset of 1,098 individuals (45%) younger than seven years of age, the NIH criteria showed sensitivity of 58.2% and specificity of 88.6% for the diagnosis of a PV in *NF1* or *SPRED1* (groups lumped together). The positive predictive value (PPV) for the NIH criteria was 80.5%, and the negative predictive value (NPV) 71.5%. At ≥7 years, the sensitivity of the NIH criteria increased to 85.3%, specificity was 74.3%, PPV 75.8% and NPV 84.3%. To increase the diagnostic rate in the age group <7 years and in the subgroup with only CALMs and skinfold freckling (with or without a family history) and no other clinical criteria, molecular diagnosis could be considered to confirm a diagnosis of NF1 or LGSS.

Clinicians should attend closely to typical pigmentary findings to help distinguish between NF1 and mosaic NF1. Participants recommended adding a clarification to the revised criteria indicating that at least one of the two pigmentary findings (CALM/freckling) should be bilateral. This should help reduce, but will not completely eliminate, the misdiagnosis of NF1 in an individual with segmental or generalized mosaic NF1. However, it highlights the possibility of mosaic NF1 when pigmentary findings are localized to one side of the body. In addition, CALMs or freckling may not be seen in older individuals, in those with many cutaneous neurofibromas, or in individuals with spinal neurofibromatosis.^28^ The diagnosis is confirmed by genetic testing or the finding of Lisch nodules/choroidal anomalies.

### Choroidal abnormalities

Choroidal abnormalities were added as an ophthalmologic criterion because of the high specificity and sensitivity for NF1^19,29^ and for the ability to differentiate NF1 from LGSS.^19^ In the revised diagnostic criteria, either Lisch nodules or choroidal abnormalities is sufficient for this criterion; choroidal abnormalities were not included as a separate criterion since isolated ophthalmologic findings, even if bilateral (e.g., an individual with *only* two Lisch nodules and two choroidal abnormalities) are likely to reflect mosaic NF1 rather than constitutional NF1.

### Affected siblings and offspring no longer qualify as criterion for NF1

Participants recommended changing “A *first-degree relative* (parent, sibling, or offspring) with NF1 by the above criteria” to “a *parent* with NF1 by the above criteria." Sibling was deleted because if only siblings are affected, one should consider a diagnosis of CMMRD.^30^ Offspring was omitted because if an adult person only has one criterion aside from an offspring fulfilling diagnostic criteria, then mosaic NF1 should be suspected in this individual. Having a parent with NF1 by the above criteria will correctly diagnose most offspring presenting with one of the other diagnostic criteria as having NF1, although, occasional co-occurrence of more than one distinct *NF1/SPRED1* pathogenic variant in a family has been reported.^31^

### Genetic concepts of importance for revised diagnostic criteria

#### Criteria for pathogenicity of variants

Per recommendations by the Human Genome Variation Society (HGVS), the term “mutation” has been replaced by the more neutral term “variant.”^32^ These variants and interpretation of variants are classified following criteria as established by their professional society. The standards and guidelines developed by the American College of Medical Genetics and Genomics (ACMG), the Association for Molecular Pathology (AMP), and the College of American Pathologists (CAP) have now been implemented widely in laboratories offering clinical molecular testing.^33^ Variants can be classified as benign (B), likely benign (LB), of uncertain clinical significance (VUS), likely pathogenic (LP), or pathogenic (P).^33^ This framework improves the interlaboratory consistency of classification and allows for *re*classification of a variant once more data have become available. The term *likely* pathogenic refers to those variants considered to have greater than 90% certainty to be disease causing. It is expected, as our understanding of *NF1* genetic variants increases, that the algorithms to classify the variants will further be improved and refined, which is especially important for those variants currently classified as VUS.

#### Pathogenic *NF1* variants

*NF1* PVs can be located across the entire coding region as well as across noncoding regions and include microdeletions spanning the *NF1* gene and multiple flanking genes; smaller intragenic copy-number variants; frameshift, nonsense and missense variants; splice site, exonic, and deep intronic variants affecting normal splicing; in-frame deletions or duplications of one to several codons; variants affecting the translational start codon; complex insertion/deletion variants; (balanced) translocations; and Alu/LINE insertions.^34^

A comprehensive approach, using dosage analysis to detect copy-number variants, and *DNA*-based sequencing, detects a PV in ~90% of classic (i.e., having pigmentary features as well as neurofibromas) well characterized nonfounder (i.e., second generation) NF1 patients. Detection rate and specificity is increased to 95–97% when an RNA-based sequencing approach, in addition to dosage analysis, is applied.^34^ Detection rates will vary in oligosymptomatic individuals or individuals with atypical presentation.^14,35^ Since genetic testing for *NF1* PV is heavily biased toward individuals with some aspect of the known NF1 phenotype we might be missing individuals with very atypical presentations. No *NF1* inactivating variants have been reported in the alternatively spliced exons of NF1 because these might be associated with no or a different phenotype. Scrutiny of genome or exome sequencing data in large cohorts of carefully phenotyped patients might shed light on this issue.

### NF1 PV alone is not sufficient for diagnosis

Diagnosis of NF1 is confirmed when an *NF1* PV is identified in an individual/fetus having either one or more of the other diagnostic criteria fulfilled. As panel testing by next-generation sequencing and exome/genome sequencing analysis is ordered with increasing frequency in individuals with a variable set of clinical features, some individuals have been found to carry an *NF1* variant (P, LP,VUS) in unaffected tissue such as blood, although NF1 was not clinically suspected. NF1 experts agreed that identification of an *NF1* variant alone does not suffice to make a diagnosis of NF1 but does require further clinical and genetic evaluation: the variant must be confirmed as pathogenic using an orthogonal method; further genetic analysis is required to verify if the variant is present as a *constitutional* (germline), *mosaic* (which may result in a mild phenotype), or *somatic* variant (e.g., due to clonal expansion in the hematopoietic stem and progenitor cells of indeterminate potential or secondary to therapy, hematological malignancy, premalignancy, or circulating tumor cells^36^). Since only one other criterion is required for a diagnosis of NF1 the *NF1* variant has to be pathogenic to fulfill the second criterion for diagnosis. Including likely pathogenic variants in the diagnostic criteria will unnecessarily reduce specificity.

Somatic second hit *NF1* PVs are typically identified in every *NF1*-associated tumor such as cutaneous and plexiform neurofibromas as well as in tissues from distinctive nontumor lesions such as CALMs,^37^ thereby resulting in biallelic *NF1* activation (Supplementary Fig. 4).

### Genetic mosaicism: mechanism, phenotypes, and implications for genetic testing

As many as 30–50% of NF1 patients are sporadic or founder patients, meaning they did not inherit the disorder from an affected parent.^1,2,8^ It is important to raise awareness that a proportion of founder patients will have the disorder due to an *NF1* variant acquired in a specific cell *after* fertilization.

These mosaic patients therefore carry the first hit *NF1* variant only in a subpopulation, as opposed to all body cells, unlike in patients with constitutional NF1 where the *NF1* variant is present in the fertilized egg. The risk for transmission to the next generation is <50% for an individual with mosaic NF1; however, if the *NF1* variant is transmitted to the next generation, offspring will carry the variant in the germline and the overall clinical presentation will usually be more severe. Depending on the timing and types of progenitor cells affected externally visible features may include pigmentary features only, cutaneous or plexiform neurofibromas only, or a combination of both, and affected body regions may range from multiple body regions crossing the midline that may resemble a generalized phenotype, to a single segment of the body not crossing the midline.^38^ Pure gonadal mosaicism, with the somatic *NF1* PVs present only in the gonads, is, however, rare.^39^

Mosaic NF1 in a patient is confirmed when an individual with features of NF1 carries a heterozygous *NF1* PV in an unaffected tissue such as blood but in significantly less than 100% of cells (variant allele fraction [VAF] < 50%). Mosaic NF1 is also confirmed if an identical first hit pathogenic *NF1* variant is identified in two or more anatomically unrelated affected lesions in the absence of this PV in unaffected tissue such as blood.

Detection of the causal *NF1* PVs in individuals with a mosaic/segmental phenotype requires special attention to (1) the sensitivity of the technology used to detect variants, as well as (2) the type of cells to be analyzed in affected tissue if the variant is not detectable in blood, i.e., melanocytes (but not keratinocytes or fibroblasts) from CALMs^37^ or Schwann cells from the cutaneous or plexiform neurofibromas.^40^

It is the responsibility of the laboratories to define and report the criteria used for the orthogonal confirmation of the variants as either constitutional/germline or mosaic.

### Orthopedic criteria

In the 1987 criteria, the orthopedic criteria included “thinning of the long bone cortex with or without pseudarthrosis.” In 2007, rewording of this criterion was suggested^25^ since thinning of long bone cortex is not the primary lesion. Rather, most children present with anterolateral bowing of the lower limb and on X-ray have medullary canal narrowing and cortical thickening at the apex of the curve in the tibia and/or fibula.^25^ The bowing may or may not progress to fracture and pseudarthrosis. Other long bones have been reported with pseudarthrosis but this is rare.

### Proposed features that were not included in the revised criteria

Some possible diagnostic criteria were not incorporated into the revised criteria because of insufficient data on specificity and/or sensitivity or other reasons. These criteria include FASI detected by MRI (given concerns about their specificity and their disappearance over time in some patients, the potential complications of anesthesia in children in scans done for diagnostic evaluation), nevus anemicus, and juvenile xanthogranuloma (Supplementary Fig. 5; both features difficult to diagnose clinically for nondermatologists and xanthogranuloma only present transiently). In addition, participants decided not to include a comment on different possible locations of skinfold freckling or comments on spinal neurofibromas. Moving forward, it will be important to collect prospective data on the diagnostic sensitivity and specificity of these clinical findings for diagnosis.

### Using the revised diagnostic criteria

It should be emphasized that clinicians do not necessarily need to search for each of the clinical features described in the criteria. For example, it is not recommended to perform biopsies or examinations under general anesthesia only to confirm diagnostic criteria. In the absence of molecular testing, serial observation will confirm whether NF1 is present in most affected individuals. In addition, one should be cautious to diagnose NF1 if neither CALMs nor neurofibromas are present, because that would be suggestive of mosaic NF1 or another unusual situation.

### Revising the nomenclature of NF1

The term “neurofibromatosis” was derived from “neurofibroma,” the term used by von Recklinghausen to describe the benign nerve sheath tumor that is the hallmark of NF1.^2^ In 2007, an NF1-like syndrome was linked to *SPRED1* PVs.^13^ Subsequent authors were concerned that any reference to NF1 in the name may confuse parents and proposed the eponymous name Legius syndrome, which has been widely adopted. During the discussions, we considered having it as a NF1 subtype but, in the absence of neurofibromas, it is inappropriate to consider it a type of neurofibromatosis.

The aim of the revision process was to help professionals distinguish between NF1 and LGSS early in life. Experts did not endorse renaming NF1 since virtually all individuals with NF1 develop neurofibromas and since the name is established by use and history. Ultimately for the syndrome associated with *SPRED1*, experts agreed that nomenclature based on nosology or pathogenesis did not improve understanding of the condition and suggested retaining the name Legius syndrome since the term is established and not misleading. We understand that a more appropriate nomenclature is needed to rename neurofibromatosis type 1, Legius syndrome, neurofibromatosis type 2, and schwannomatosis. This will be one of the future tasks of the steering committee.

### Continued revision of the diagnostic criteria

The proposed criteria for diagnosis of NF1 and LGSS represent the first coordinated attempt by our community to update the diagnostic criteria since 1987. Given the challenge in organizing the input of multiple experts, patients, and advocacy groups in this process, it is unlikely that a major revision to the criteria will be proposed in the near future. However, it is likely that refinement of these diagnostic criteria will be necessary in the future. For this reason, the CTF will sponsor an ongoing initiative to evaluate and recommend proposed changes to the diagnostic criteria for NF1 and LGSS. We anticipate that this group of experts will meet periodically to solicit input from the community, to review data relevant to diagnostic criteria, and will publish its consensus recommendations periodically for use by the larger community.

## Supplementary information

Supplementary Information

## Data Availability

The data from the modified Delphi process are available in the supplementary data and figure section.
