# Supplementary material for: Revised diagnostic criteria for neurofibromatosis type 1 and Legius syndrome: an international consensus recommendation
Source: Genet Med. 2021 May 19;23(8):1506–13. doi: 10.1038/s41436-021-01170-5 (PMC8354850; doi:10.1038/s41436-021-01170-5)
Supplement: Supplementary file 1 — Supplementary Information [file 41436_2021_1170_MOESM1_ESM.pdf]

**Supplementary table 1:** Current diagnostic criteria for neurofibromatosis type 1 and issues reviewed by the consensus panel.

| **EXISTING NF1 DIAGNOSTIC CRITERIA (The diagnostic criteria for NF1 are met in an individual if two or more of the following are present)** | **ISSUES REVIEWED BY CONSENSUS PANEL** |
|---|---|
| Six or more café-au-lait macules over 5 mm in greatest diameter in prepubertal individuals and over 15 mm in greatest diameter in post - pubertal individuals | • Current pigmentary criterion fails to distinguish among NF1, mosaic NF1, Legius syndrome, and mosaic Legius syndrome |
| Freckling in the axillary or inguinal region | • Current pigmentary criterion fails to distinguish among NF1, mosaic NF1, Legius syndrome, and mosaic Legius syndrome<br>• Presence of unilateral pigmentary changes should raise suspicion for mosaic NF1 |
| Two or more neurofibromas of any type *or* one plexiform neurofibroma | • No issues |
| Optic glioma | • Minor wording change to “optic pathway glioma” |
| Two or more Lisch nodules (iris hamartomas) | • No issues |

| | |
|---|---|
| A distinctive osseous lesion such as sphenoid dysplasia or thinning of long bone cortex with or without pseudarthrosis | • Pseudarthrosis is usually preceded by congenital bowing of a long bone and only rarely by thinning of the cortex<br>• Congenital bowing is usually associated with thickening of the cortex of the long bone<br>• Naming the long bones would be more specific and attract attention of the clinician to the proper clinical examination of these body areas |
| A first-degree relative (parent, sibling, or offspring) with NF1 by the above criteria | • Use of siblings might result in possible incorrect diagnosis of NF1 in case of constitutional mismatch repair deficiency (CMMRD) syndrome<br>• Use of offspring might result in possible diagnosis of NF1 in a parent with mosaic NF1 |
| **ADDITIONAL FEATURES OF NF1** | **ISSUES REVIEWED BY CONSENSUS PANEL** |
| Dystrophic scoliosis | • Dystrophic scoliosis is typical of NF1 and should be clearly defined |
| Juvenile xanthogranuloma | • Multiple juvenile xanthogranulomas are not rare in NF1 and are present in very young children in up to 30% of cases. The lesion has not been reported in Legius syndrome |

| | |
|---|---|
| Nevus Anemicus | • Nevus anemicus can be present in up to 50% of young children with NF1 and would allow a clinical diagnosis at a young age but they can also be seen in other conditions and in the general population |
| Choroidal Anomalies | • Choroidal abnormalities are seen in up to 60-70% of children with NF1 |
| Focal areas of signal intensity (FASI) | • FASI are common findings on MRI scan of NF1 patients; they may disappear with time and require MRI with sedation in young children |

# Supplementary figure 1

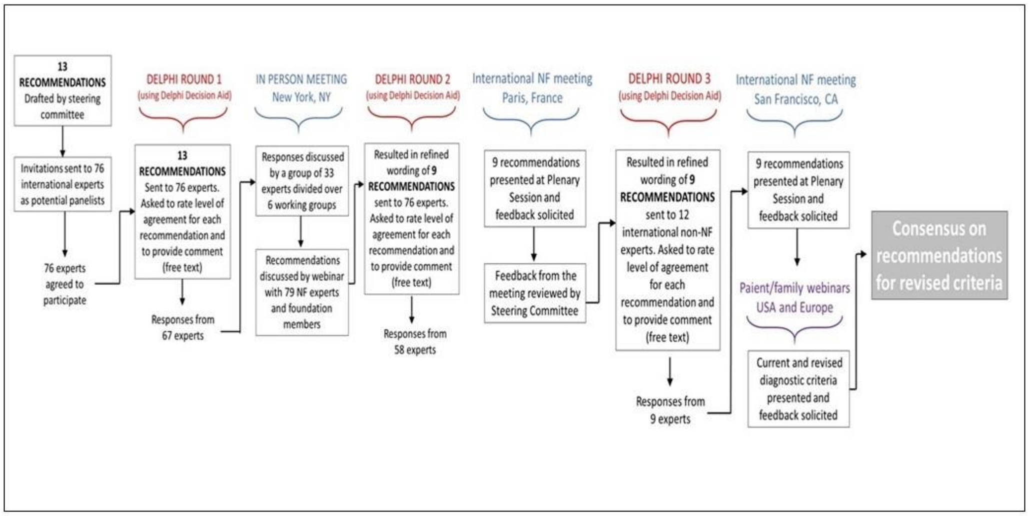


Schematic of the modified Delphi process used to develop revised diagnostic criteria for NF1.

# Supplementary figure 2

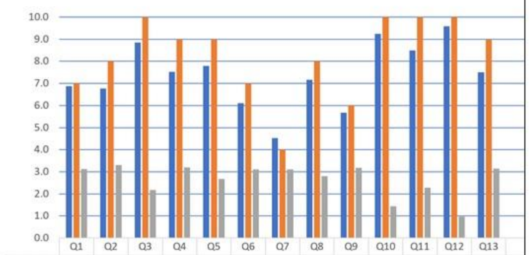

| | Q1 | Q2 | Q3 | Q4 | Q5 | Q6 | Q7 | Q8 | Q9 | Q10 | Q11 | Q12 | Q13 |
|---|---|---|---|---|---|---|---|---|---|---|---|---|---|
| Average | 6.9 | 6.8 | 8.9 | 7.5 | 7.8 | 6.1 | 4.5 | 7.2 | 5.7 | 9.2 | 8.5 | 9.6 | 7.5 |
| Median | 7.0 | 8.0 | 10.0 | 9.0 | 9.0 | 7.0 | 4.0 | 8.0 | 6.0 | 10.0 | 10.0 | 10.0 | 9.0 |
| SD | 3.1 | 3.3 | 2.2 | 3.2 | 2.7 | 3.1 | 3.1 | 2.8 | 3.2 | 1.4 | 2.3 | 1.0 | 3.1 |

**NF1**

**Q1** first degree relative
**Q2** lump CAL - freckling
**Q3** bowing long bones
**Q4** naming long bones
**Q5** add dystrophic scoliosis
**Q6** >=2 JXG
**Q7** nevus anemicus
**Q8** >=2 choroidal noduli
**Q9** MRI T2 hyperintensities
**Q10** pathogenic mutation
**Q11** define mosaic NF1
**Q12** keep other criteria
**Q13** NF1+ Legius// NF2 + Schw

Results of first Delphi process for NF1. Q1-13 refers to the questions of the Delphi questionnaire. SD: standard deviation; Average: average score on a scale going from 0 to 10 (do not agree – agree); Median: median score on scale from 0 to 10. CAL: café-au-lait; >=2 JXG: 2 or more juvenile xanthogranulomas ; NF1 + Legius// NF2 + sch : keep NF1 and Legius syndrome in one group and NF2 and schwannomatosis in another group.

# Supplementary figure 3

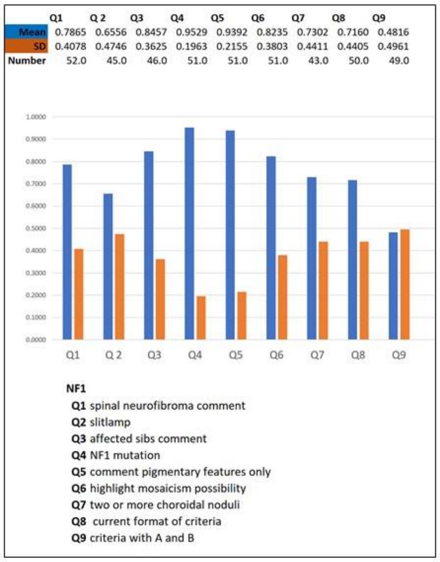

| | Q1 | Q 2 | Q3 | Q4 | Q5 | Q6 | Q7 | Q8 | Q9 |
|---|---|---|---|---|---|---|---|---|---|
| Mean | 0.7865 | 0.6556 | 0.8457 | 0.9529 | 0.9392 | 0.8235 | 0.7302 | 0.7160 | 0.4816 |
| SD | 0.4078 | 0.4746 | 0.3625 | 0.1963 | 0.2155 | 0.3803 | 0.4411 | 0.4405 | 0.4961 |
| Number | 52.0 | 45.0 | 46.0 | 51.0 | 51.0 | 51.0 | 43.0 | 50.0 | 49.0 |



Results of second Delphi process for NF1. Q1-9: refers to the questions of the Delphi questionnaire. SD: standard deviation; mean : mean score on questions with possible answers 0 = do not agree and 1 = agree.

# Supplementary figure 4

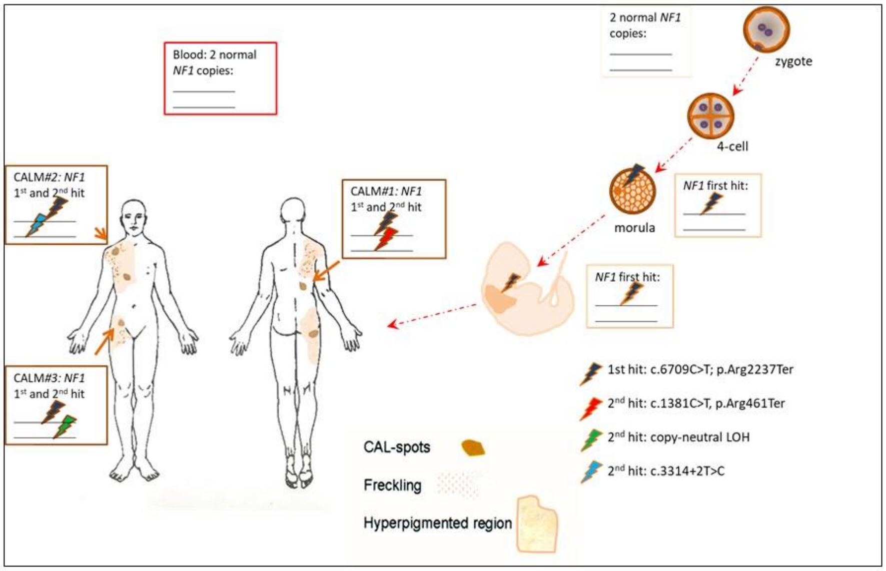


**Segmental/localized or generalized mosaic NF1 is caused by somatic *NF1* pathogenic variants.** Example of genetic testing results in a segmental/localized mosaic patient with pigmentary lesions-only. Three biopsies (2-mm punch biopsies) indicated on the body diagram were taken and after *in vitro* selective culture of melanocytes subjected to comprehensive RNA-based *NF1/SPRED1* genetic testing. In the patient here discussed, genetic testing from melanocytes, cultured from the CALMs, showed a common somatic *NF1* first hit in all biopsied CALMs, which was absent in the blood. In addition, a different somatic *NF1* "second hit" pathogenic variant was found in the melanocytes from every CALM, confirming the diagnosis of segmental/mosaic localized NF1.

# Supplementary figure 5

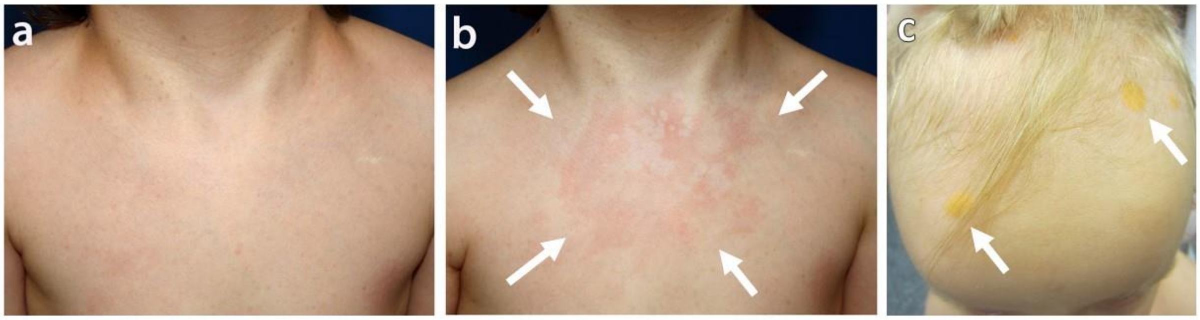


**Dermatologic manifestations of NF1 not included in the revised diagnostic criteria**

(Panel a) Nevus anemicus indistinguishable from normal skin.

(Panel b) Appearance of the nevus anemicus with characteristic area of pale skin after rubbing the skin.

(Panel c): juvenile xanthogranulomas in a child with neurofibromatosis 1. Note the distinctive yellow appearance of the lesions.

# Supplementary figure 6

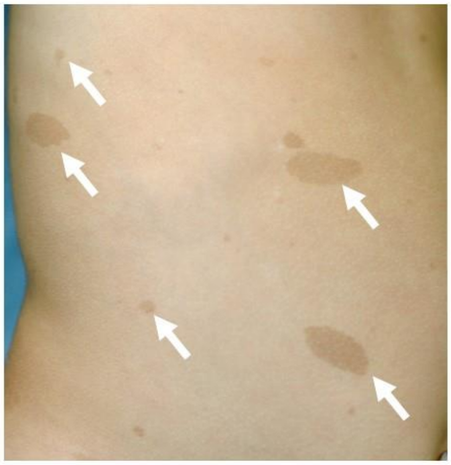

**Dermatologic manifestations of NF1**

Multiple CALMs of more than 5 mm in a child with NF1

# Supplementary figure 7: Freckling

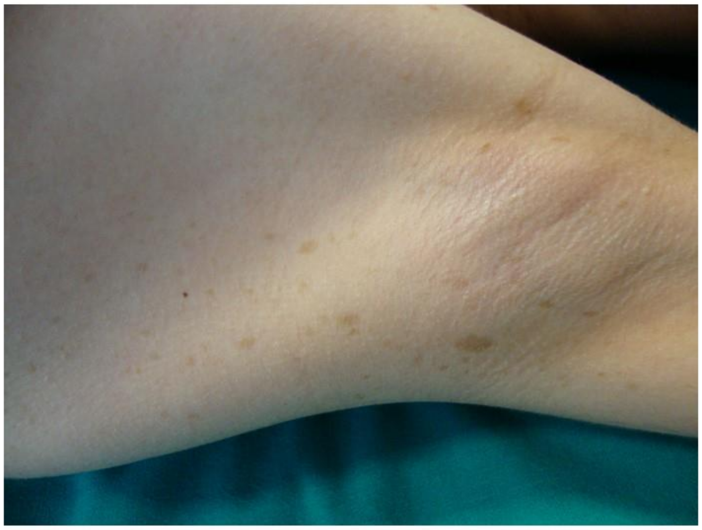

**Dermatologic manifestations of NF1**

Axillary freckling in a child with neurofibromatosis 1.

# Supplementary figure 8a: Cutaneous neurofibromas

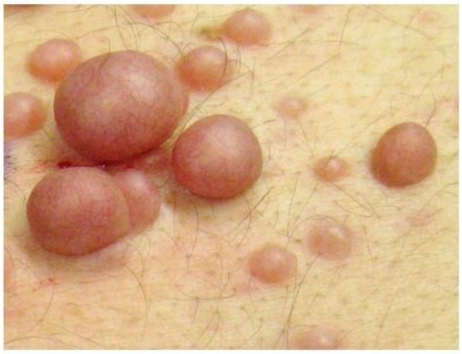

**Neurofibromas in a person with NF1**

(Panel A): Numerous cutaneous neurofibromas in an adult with neurofibromatosis 1.

# Supplementary figure 8b: (plexiform NF)

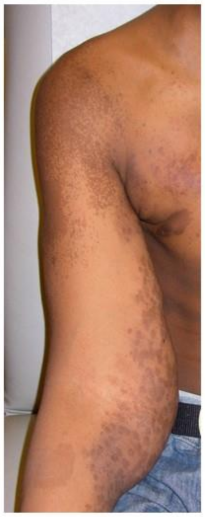

(Panel B) Plexiform neurofibroma of the right arm in an adolescent with neurofibromatosis 1.

# Supplementary figure 9

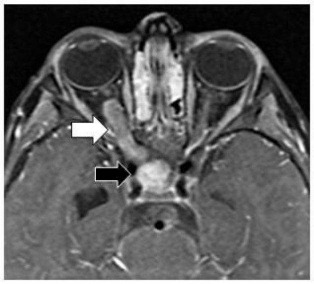

**Optic pathway glioma in person with NF1**

MRI scan of brain showing glioma of right optic nerve (white arrow) and glioma of chiasm with contrast enhancement (black arrow).

Supplementary figure 10a:
Lisch nodules of the iris

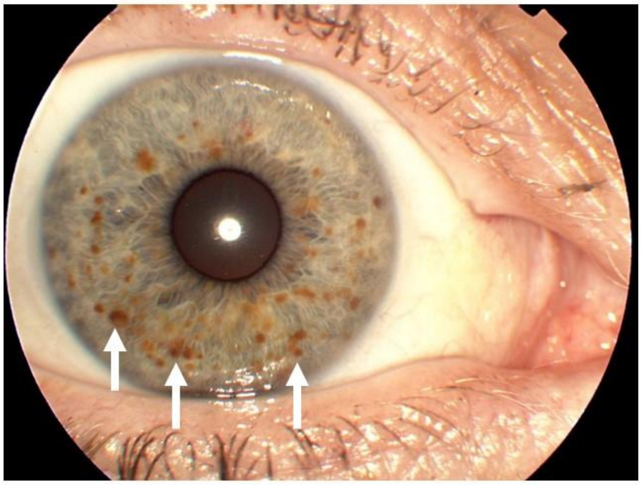

**Ophthalmologic features used for diagnosis of NF1**

(Panel A) Photograph of an eye of a person with NF1 demonstrating multiple Lisch nodules of the iris (white arrows).

# Supplementary figure 10b: Choroidal abnormalities

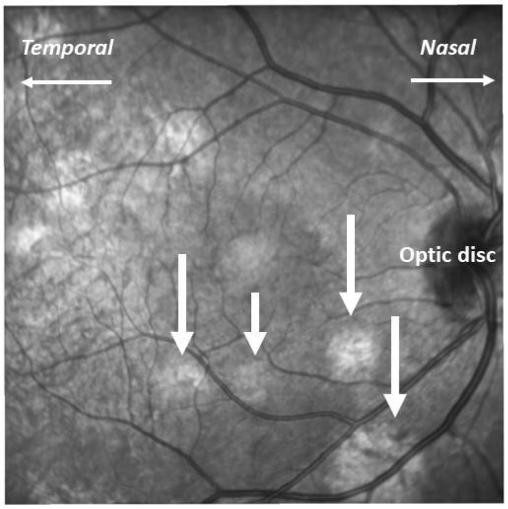


(Panel B) infrared imaging of the eye of a person with NF1 demonstrating multiple hyperreflective lesions in the choroid consistent with choroidal abnormalities (arrowheads).

# Supplementary figure 11

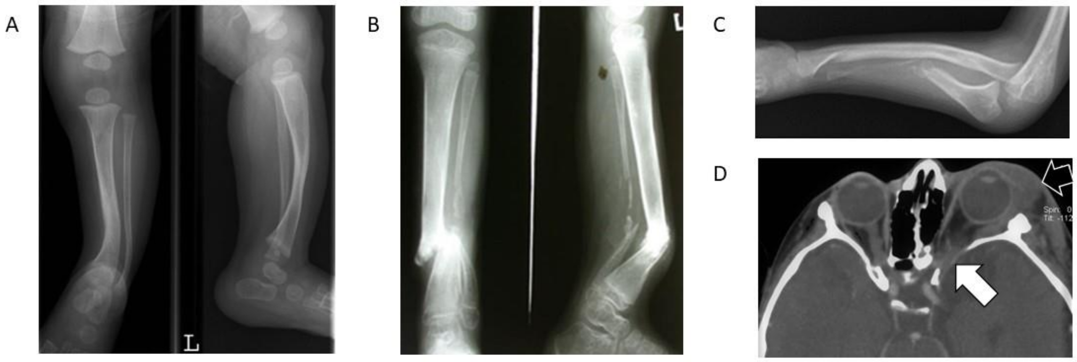


**Skeletal features of NF1**

(Panel A) X-ray of the left lower leg: typical antero-lateral bowing of tibia and fibula; (Panel B) X-ray of left lower leg: pseudarthrosis of tibia and fibula;  (Panel C) X-ray of forearm showing pseudarthrosis of ulna and bowing of radius; (Panel D) CT scan showing sphenoid bone dysplasia (white arrow) and orbital plexiform neurofibroma (black arrow). Note that sphenoid bone dysplasia in combination with orbital plexiform neurofibroma counts for only one diagnostic criterion.

# Supplementary figure 12

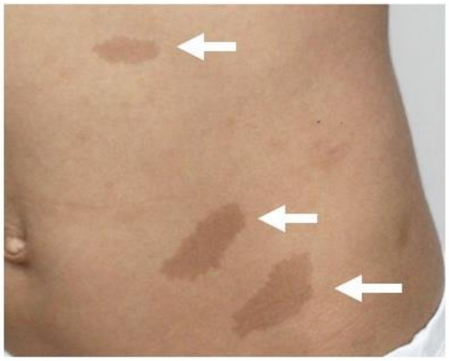

**Dermatologic manifestations of Legius syndrome**

Typical café-au-lait macules in patients with Legius syndrome

**Supplementary introduction**.

Descriptions of individuals purported to have NF1 have been found in manuscripts dating from 1000 or earlier (1). However, it was not until 1881 that von Recklinghausen identified the nerve origin of the tumors and coined the term ‘‘neurofibroma’’ to describe benign tumors arising from the peripheral nerve sheath (2). His colleagues honored his contribution by naming the condition von Recklinghausen’s disease. The first report of NF2 was by Wishart in 1822 (3), who described a patient with severe manifestations of NF2. Subsequent reports were mixed as to whether acoustic neuromas were part of NF1 or a separate condition. Harvey Cushing concluded they were part of the NF1 spectrum in his monograph on acoustic neuromas (4). Perhaps as a result of Cushing’s eminence, most subsequent authors lumped the conditions together as neurofibromatosis or von Recklinghausen disease. Thus, Crowe and colleagues reported that 5% of cases in their large study had acoustic neuromas (5). Even in the early era of CT scanning, optic meningiomas were confused with optic gliomas and the wrong kind of NF diagnosed.

Despite the confusion with NF2, the literature in the first half of the twentieth century reported the characteristic disease features of NF1. In 1896, CALMs were described in two French publications, and in 1901, Adrian noted the high incidence of skeletal changes such as scoliosis. In 1937, Lisch clarified the association with pigmented iris nodules, initially described by Waardenburg in 1918. In 1940, Davis established optic pathway glioma as a feature of NF1. In 1956, Crowe determined the diagnostic importance of CALMs and axillary freckling (6, 7) and the study of Borberg (8) in Denmark helped establish the disease spectrum.

1. Ruggieri, M., et al., Early history of the different forms of neurofibromatosis from ancient Egypt to the British Empire and beyond: First descriptions, medical curiosities, misconceptions, landmarks, and the persons behind the syndromes. Am J Med Genet A, 2018. 176(3): p. 515-550.

2. von Recklinghausen, F.D., Uber Die Multiplen Fibrome der Haut und Ihre Beziehung zu den Multiplen Neuromen. 1882, Berlin: Hirschwald.

3. Wishart, J.H., Case of tumours in the skull, dura mater, and brain. Edinburgh Med Surg J, 1822. 18: p. 393-397.

4. Cushing, H., Bilateral tumors and generalized neurofibromatosis. 1917, Hafner Publishing Company: New York. p. 210-216.

5. Crowe, F.W., W.J. Schull, and J.E. Neely, A clinical pathological and genetic study of multiple neurofibromatosis. American Lecture Series in Dermatology. 1956, Springfield, IL: Charles C. Thomas. 181.

6. Crowe, F.W. and W.J. Schull, Diagnostic importance of cafe-au-lait spot in neurofibromatosis. AMA Arch Intern Med, 1953. 91(6): p. 758-66.

7. Crowe, F.W., Axillary Freckling as a Diagnostic Aid in Neurofibromatosis. Ann Intern Med, 1964. 61: p. 1142-3.

8. Borberg, A., Clinical and genetic investigations into tuberous sclerosis and Recklinghausen's neurofibromatosis; contribution to elucidation of interrelationship and eugenics of the syndromes. Acta Psychiatr Neurol Scand Suppl, 1951. 71: p. 1-239.

**Supplementary Materials and Methods**

A steering committee of seven people reviewed the literature and generated 13 statements (supplementary data 1) regarding potential changes to the diagnostic criteria for NF1. A group of 76 international experts in the field and reflecting the cross section of specialties involved in NF1 diagnosis were identified to participate in the modified Delphi procedure using the Delphi Decision Aid freeware program developed by J. Scott Armstrong (http://scott.armstrong.delphi.stlouisintegration.com/delphi2/). Experts were invited to provide a score from 0 (no agreement) to 10 (complete agreement) for each statement concerning revision of the diagnostic criteria for NF1. Participants could choose not to score a statement. They were also invited to explain their score using free text. All scores were recorded anonymously by the software. The first set of 13 statements was scored in May, 2018 and all participants were provided with all summary scores and free text comments.

The results of the first Delphi were discussed by a group of 33 experts divided over six working groups at a meeting in NYC, USA on June 11-12, 2018 (supplementary data 2). This meeting was followed by a webinar on August 31, 2018, attended by 79 people (experts and administrators of CTF) to determine the level of consensus for the initial statements. Based on the results of the first round of Delphi statements, the in-person meeting in NYC, and the feedback during the webinar, the steering committee designed a set of nine revised statements (supplementary data 3) for a second round of Delphi (with same instructions as noted above). The second set of statements was scored in October, 2018 and all participants received feedback on the summary scores and free text comments.

These results were presented during a plenary session at the Joint Global Neurofibromatosis

Conference in Paris, France on November 6, 2018, and attendees were invited to provide feedback on the CTF website. The proposed diagnostic criteria were further revised according to this feedback. In April, 2019, a group of 13 non-NF specialists from different parts of the world were asked about the ease of use of the proposed diagnostic criteria (supplementary data 5).

The Steering Committee actively sought input from patients, families, and advocates regarding the proposed criteria and changes to nomenclature through a series of webinars in June and July, 2019, in the United States and Europe. On September 21, 2019, the revised diagnostic criteria were presented for the last round of feedback at the 2019 NF Conference in San Francisco, CA, USA, during the Clinical Care Symposium. Attendees again had the option to leave comments on the CTF website. The final diagnostic criteria were established in January 2020.

**Supplementary data 1**. Questions were generated by the steering committee (DG. Evans, S. Huson, E. Legius, L. Messiaen, P. Pancza, S. Plotkin, P. Wolkenstein) and presented to experts in the first Delphi process.

Question 1: NF1 clinical working group

CURRENT CRITERIA:

- A first-degree relative (parent, sibling, or offspring) with NF1 by the above criteria.

NEW CRITERIA SUGGESTION: to drop sibling and offspring from criteria

- (drop sibling and offspring) *A parent with NF1 by the above criteria

RATIONALE:

Use of sibs might result in possible diagnosis of NF1 in case of constitutional mismatch repair deficiency syndrome in siblings (autosomal recessive condition with severe phenotype; Wimmer et al Clinical Genetics 2017); use of offspring might result in possible diagnosis of NF1 in a mosaic parent.

Provide arguments for or against including references if relevant.

Question 2: NF1 dermatology/clinical working groups

CURRENT CRITERIA:

- *Six or more café-au-lait macules over 5 mm in greatest diameter in prepubertal individuals and over 15 mm in greatest diameter in post - pubertal individuals.
- *Freckling in the axillary or inguinal region.

NEW CRITERIA SUGGESTION: only one criterion remains

- *Six or more café-au-lait macules over 5 mm in greatest diameter in prepubertal individuals and over 15 mm in greatest diameter in postpubertal individuals, with or without freckling in the axillary or inguinal region.

RATIONALE:

Combining the two pigmentary criteria into one criterion reduces the chance of a person with Legius syndrome to be diagnosed as NF1. Loss of sensitivity may be compensated by adding additional criteria that are present at a young age such as nevus anemicus, juvenile xanthogranuloma, choroidal noduli, neuroradiological abnormalities or a constitutional pathogenic *NF1* variant. Provide arguments for or against, including references if relevant.

Question 3: NF1 skeletal working group

CURRENT CRITERIA:

- A distinctive osseous lesion such as sphenoid dysplasia or thinning of long bone cortex with or without pseudarthrosis.

NEW CRITERIA SUGGESTION: replace thinning of long bone by congenital bowing of a long bone

- A distinctive osseous lesion such as sphenoid dysplasia or congenital bowing of a long bone with or without pseudarthrosis.

RATIONALE:

Pseudarthrosis in NF1 is usually preceded by congenital bowing of a long bone and only rarely by thinning of the cortex. Congenital bowing is usually associated by thickening of the cortex of the long bone. (Stevenson DA, Genet Med. 2007 Jul;9(7):409-12.) Provide arguments for or against, including references if relevant.

Question 4: NF1 skeletal working group

CURRENT CRITERIA:

- A distinctive osseous lesion such as sphenoid dysplasia or thinning of long bone cortex with or without pseudarthrosis.

NEW CRITERIA SUGGESTION: naming the long bones

- A distinctive osseous lesion such as sphenoid dysplasia or congenital bowing of a long bone (tibia, fibula, ulna, clavicle and radius) with or without pseudarthrosis.

RATIONALE: naming the long bones is more specific. It also attracts attention of the clinician to the proper clinical examination of these body areas. Provide arguments for or against, including references if relevant.

Question 5: NF1 skeletal working group

CURRENT CRITERIA:

- A distinctive osseous lesion such as sphenoid dysplasia or thinning of long bone cortex with or without pseudarthrosis.

NEW CRITERIA SUGGESTION: adding dystrophic scoliosis

- *A distinctive osseous lesion such as sphenoid dysplasia, dystrophic scoliosis or congenital bowing of a long bone (tibia, fibula, ulna, clavicle and radius) with or without pseudarthrosis.

RATIONALE: Dystrophic scoliosis should be clearly defined but it is typical of NF1 and increases the sensitivity of the criteria (more children will fulfill criteria at a younger age) by a small percentage. Provide arguments for or against, including references if relevant.

Question 6: NF1 dermatology working group

ADDITIONAL CRITERIA SUGGESTION:

- *Two or more juvenile xanthogranuloma at present or in the past.

RATIONALE:

Multiple juvenile xanthogranuloma are not rare in NF1 and are present in very young children in up to 30% of cases. This may again increase the sensitivity of the criteria and increase the number of cases that fulfill criteria at a young age. The lesion can be reliably diagnosed clinically by a dermatologist or an experienced NF1 expert without biopsy. (Fenot et al., J Am Acad Dermatol. 2014 Aug;71(2):389-90). The lesion has not been reported in Legius syndrome. Provide arguments for or against, including references if relevant.

Question 7: NF1 dermatology working group

ADDITIONAL CRITERIA SUGGESTION

- *At least one nevus anemicus

RATIONALE:

Nevus anemicus can be present in young children and would allow a clinical diagnosis of NF1 at a young age in the group with this sign. It is reported to be seen in up to half of the children with

NF1 (Marque et al., J Am Acad Dermatol 2013;69:768-75; Tadini et al., Dermatology 2013;226:115-118; Hernández-Martín et al., Pediatr Dermatol 2015). It is however not specific for NF1 and is also seen in the general population (2%) and in other RASopathies (rarely in Noonan syndrome with multiple lentigines and Legius syndrome; Bulteel et al., J Am Acad Dermatol, 2018). Please comment on the size or other special aspects of these lesions in NF1. Provide arguments for or against, including references if relevant.

Question 8: NF1 ophthalmology working group

ADDITIONAL CRITERIA SUGGESTION:

- Minimum 2 choroidal noduli in one eye seen by infrared optical coherence tomography (OCT)

RATIONALE:

Choroidal abnormalities are seen in up to 60-70% of children with NF1 and the examination can be done from the age of 5 years on (Parrozzani et al., Invest Ophthalmol Vis Sci. 2015 Sep;56(10):6036-42. Sensitivity, specificity, and positive and negative predictive values of NF1related choroidal abnormalities were 0.60, 0.97, 0.98, and 0.46, respectively). It is more frequent in children than Lisch noduli (Viola et al., Ophtalmology 2012/ Vagge et al. , Acta Ophthalmol 2015). This sign seems to be specific for NF1 and is not seen in Legius syndrome (Cassiman et al., Clin Genet 2016/ Tucci et al., J Hum Genet, 2017). The OCT device is broadly available in eye clinics. Provide arguments for or against, including references if relevant.

Question 9: NF1 neuroradiology working group

ADDITIONAL CRITERIA SUGGESTION:

- Two or more brain MRI T2 hyperintensities (focal areas of signal intensity; FASI) in the cerebellum, brainstem or basal ganglia.

RATIONALE:

Could be an early sign of NF1 in children undergoing a brain MRI. (Sabol et al., Croat Med J, 2011, The diagnostic sensitivity, specificity, and accuracy rate of T2-hyperintensities for NF1 were highest in the youngest age (2-7 years): 81% (95% CI 71%-89.1%), 99% (95% CI 92.3%100%), and 85.8 (95% CI 83.3-93.8), respectively.) See also DeBella et al., Neurology 2000. Provide arguments for or against, including references if relevant. Please comment on number needed to fulfill criterion.

Question 10: NF1 genetics working group

ADDITIONAL CRITERIA SUGGESTION:

- *A pathogenic *NF1* variant

RATIONALE:

Increasingly more children with only CAL maculae are subjected to genetic analysis as it differentiates from Legius syndrome, CMMRD and other CAL syndromes. Pathogenicity should be determined by the proper criteria established at that moment by the professional society (Richards S. et al, Genetics in Medicine 5:403;2015). A pathogenic *NF1* variant is found in more than 90% of children and adults fulfilling NIH criteria (Messiaen et al., Human Mut 2000; Wimmer et al., Human Mut 2007; Sabbagh et al., Human Mut 2013). Detection rates vary in individuals submitted for genetic testing due to diagnostic uncertainty or atypical presentation (Messiaen et al., JAMA 2009, table 3). Provide arguments for or against, including references if relevant.

Question 11: NF1 genetics working group

- We need to define mosaic NF1 in the text accompanying the new diagnostic criteria

RATIONALE:

This is important for correct diagnosis and counseling. The risks for transmitting the *NF1* pathogenic variant to offspring are lower if one of the parents is mosaic for an *NF1* pathogenic variant. Mosaic NF1 can present as a localized (segmental) form of NF1 or as a generalized form of NF1. Mosaic NF1 should be differentiated from epidermal nevus syndrome etc. Provide arguments for or against, including references if relevant.

Question 12: NF1 clinical/dermatology/ophthalmology working groups

CURRENT CRITERIA:

The coordinating group was unanimous that the following criteria should remain unchanged, with the exception of a minor wording change in one of the three (optic glioma):

- (2) Two or more neurofibromas of any type or one or more plexiform neurofibromas.
- (3) Optic glioma (tumor of Optic pathway) SHOULD READ '*Optic pathway glioma'*)
- (5) Two or more Lisch nodules (benign iris hamartomas)

RATIONALE: These criteria have stood the test of time well with no new differential diagnoses with the same feature(s). Optic pathway glioma is now in routine use and needs no clarification. Provide arguments for or against, including references if relevant.

Question 13: NF1 clinical working group

- It is time to split NF1/Legius syndrome from the NF2/Schwannomatosis group

RATIONALE:

The biology and clinical presentation of NF1/Legius is sufficiently different from NF2/Schwannomatosis to split into two groups. Please provide arguments for or against.

**Supplementary data 2**. List of Steering Committee members and participants of Diagnostic Criteria workshop in New York City June 11-12, 2018

Steering committee:

1. D. Gareth Evans (Medical Genetics, University of Manchester, UK)
2. Susan Huson (Medical Genetics, University of Manchester, UK)
3. Eric Legius (Medical Genetics, KU Leuven, Belgium)
4. Ludwine Messiaen (Medical Genetics, University of Alabama at Birmingham, USA)
5. Patrice Pancza (Children’s Tumor Foundation, New York, USA)
6. Scott Plotkin (Neuro-Oncology, Massachusetts General Hospital, USA)
7. Pierre Wolkenstein (Dermatology, Hôpital Henri-Mondor, France)

Participants of Diagnostic Criteria Workshop

1. Robert A. Avery (Pediatric Neuro-Ophthalmology, Children’s Hospital of Philadelphia, USA)
2. Yemima Berman (Medical Genetics, University of Sydney, Australia)
3. Jaishri Blakeley (Neuro-Oncology, Johns Hopkins University Hospital, USA)
4. Dusica Babovic-Vuksanovic (Medical Genetics, Mayo Clinic, USA)
5. Karin Soares Cunha (Pathology, Universidade Federal Fluminense, Brazil)
6. D. Gareth Evans (Medical Genetics, University of Manchester, UK)
7. Rosalie Ferner (Neurology, Guy’s and St. Thomas’ Hospital and NHS Trust, UK)
8. Michael J. Fisher (Pediatric Neuro-Oncology, The Children’s Hospital of Philadelphia, USA)
9. Jan Friedman (Medical Genetics, University of British Columbia, Canada)

10. Marco Giovannini (Head and Neck Surgery, University of California, Los Angeles, USA)

11. David H. Gutmann (Neurology, Washington University, USA)

12. Susan Huson (Medical Genetics, University of Manchester, UK)

13. Michel Kalamarides (Neurosurgery, Hôpital Pitié Salpêtrière, France)

14. Hildegard Kehrer-Sawatzki (Institute of Human Genetics, University of Ulm, Germany)

15. Bruce R. Korf (Medical Genetics, University of Alabama at Birmingham, USA)

16. Eric Legius (Medical Genetics, KU Leuven, Belgium)

17. Mia MacCollin (Neurology, Bend, Oregon, USA)

18. Victor-Felix Mautner (Neurology/Psychiatry, University of Hamburg – Eppendorf, Germany)

19. Ludwine Messiaen (Medical Genetics, University of Alabama at Birmingham, USA)

20. Laura Papi (Molecular Genetics, University of Florence, Italy)

21. Sirkku Peltonen (Dermatology, University of Turku, Finland)

22. Scott Plotkin (Neuro-Oncology, Massachusetts General Hospital, USA)

23. Katherine A Rauen (Medical Genetics, University of California, Davis, USA)

24. Vincent Riccardi (Medical Genetics, Crescenta, California, USA)

25. Martino Ruggieri (Unit of Rare Diseases of the Nervous System in Childhood, Department of Clinical and Experimental Medicine, Section of Pediatrics and Child Neuropsychiatry, University of Catania, Italy)

26. Elizabeth Schorry (Medical Genetics, Cincinnati Children's Hospital Medical Center, USA)

27. Miriam J. Smith (Molecular Genetics, University of Manchester, UK)

28. Anat Stemmer-Rachamimov (Neuropathology, Massachusetts General Hospital, USA)

29. David A. Stevenson (Medical Genetics, Stanford University, USA)
30. Gianluca Tadini (Dermatology, University of Milan, Italy)
31. Nicole Ullrich (Pediatric Neuro-Oncology, Childrens Hospital of Boston, USA)
32. David Viskochil (Medical Genetics, University of Utah, USA)
33. Katharina Wimmer (Institute of Human Genetics, Medical University of Innsbruck, Austria)
34. Pierre Wolkenstein (Dermatology, Hôpital Henri-Mondor, Paris - Creteil, France)
35. Kaleb Yohay, (Pediatric Neurology, NYU Langone Health, USA)

**Supplementary data 3**. Questions posed to experts in the second Delphi process.

1. Two or more neurofibromas of any type or one or more plexiform neurofibromas or large internal neurofibromas. Some patients present with multiple bilateral spinal root neurofibromas and may not meet other criteria; consider genetic testing and/or expert opinion.

QUESTION- We have added a comment re ‘spinal NF’. Do you think this is necessary?

RATIONALE- Patients with predominantly spinal involvement may have few if any CAL macules and cutaneous neurofibromas (Messiaen et al, J Med Genet, 2003 PMID 12566521; Korf et al, N Eng J Med, 2005 PMID: 15858190; Burkitt-Wright et al, J Med Genet, 2013 PMID: 23812910).

2. Two or more iris Lisch nodules identified by slit lamp examination, or indirect ophthalmoscopy in very young children who cannot cooperate with slit lamp examination. * QUESTION- The comment re slit lamp, etc. is an addition, is it necessary?

RATIONALE- Patients are sometimes told they have nodules based on direct ophthalmoscopy and on tertiary referral are found to have iris nevi.

3. A first-degree relative (parent, sibling or child) with NF1 by the above criteria. If only two affected sibs, consider genetic testing and/or expert opinion.

QUESTION- A proviso re affected sibs has been added, the rationale would be discussed in the paper. Is this useful?

RATIONALE- The proviso was added for two reasons: a) families with constitutional mismatch repair deficiency and b) pure gonadal mosaicism is VERY rare in NF1 and affected sibs with no

FH may have different pathogenic variants. An alternative is to just use ‘a parent’ with NF1.

4. An apparent heterozygous pathogenic *NF1* variant in unaffected tissue such as white blood cells.

QUESTION - Do you agree a pathogenic *NF1* variant should be added to the criteria?

RATIONALE - Genetic testing now detects a pathogenic variant in over 90% of people with typical NF1. There are an increasing number of clinically useful genotype-phenotype correlations (Microdeletions: Recent review by Kehrer-Sawatzki, Hum Genet, 2017, PMID: 28213670; Met992del:Upadhyaya et al, Am J Hum Genet 2007, PMID: 17160901; Koczkowska et al, Genet in Medicine 2018, PMID:30190611; Arg1809: Rojnueangnit et al, Hum Mutat, 2015,

PMID:26178382; Pinna et al, Eur J Hum Genet, 2015, PMID: 25370043; AA844-848: Koczkowska et al, Am J Hum Genet 2018, PMID: 29290338). In Legius syndrome similar pigmentary changes are caused by variants in *SPRED1* (review Brems et al, Hum Mutat 2012, PMID: 22753041).

5. Bilateral freckles in axilla, groin, neck, sub mammary region in women and peri-oral region. IF diagnosis is based on pigmentary features alone consider genetic testing and/or expert opinion. *

QUESTION - A sentence of caution has been added about pigmentary features alone (only CALM and freckling). Do you think this is useful?

RATIONALE - *SPRED1* pathogenic variants have been reported in between 2 and 8% of children with >5 CAL and no FH (Messiaen et al JAMA 2009, PMID 19920235; Evans et al

EBioMedicine 2016, PMID 27322474). Genetic testing will also identify the cases with NF1 pathogenic variants that appear not to be associated with cutaneous neurofibromas.

6. Patients with somatic mosaicism for a pathogenic variant in the *NF1* gene can present with localized or segmental disease. This includes isolated plexiform neurofibromas +/- local bone changes.

QUESTION - Do you think it is necessary to highlight the possibility of mosaicism?

RATIONALE - The asterisks and footnote are added after the criteria that may give a clue to mosaic NF1 clinically (CALM, neurofibromas, Lisch nodules, freckles, pathogenic *NF1* variant). Currently non-experts may diagnose full blown NF1 if, for example, a child has multiple CAL and freckling in one part of the body.

7. Two or more choroidal abnormalities (CAs)

QUESTION - Do you agree with adding CAs to the criteria?

RATIONALE - CAs are a frequent finding in NF1 and are a useful diagnostic aid in difficult cases. It is NOT envisaged we would recommend examination if it required general anesthesia. Choroidal abnormalities are seen in up to 60-70% of children with NF1 and the examination can be done from the age of 5 years on (Parrozzani et al., Invest Ophthalmol Vis Sci. 2015 Sep;56(10):6036-42. Sensitivity, specificity, and positive and negative predictive values of NF1related choroidal abnormalities were 0.60, 0.97, 0.98, and 0.46, respectively). It is more frequent in children than Lisch nodules (Viola et al., Ophthalmology 2012; Vagge et al., Acta Ophthalmol 2015). This sign seems to be specific for NF1 and is not seen in Legius syndrome (Cassiman et al., Clin Genet 2016; Tucci et al., J Hum Genet, 2017). The OCT device is broadly available in eye clinics.

8. QUESTION - Do you think the criteria should continue with the current format? i.e. you have a list of criteria and need two or more. If the above changes are made these would look like:

NF1 would be diagnosed with any two of the following:

1. Six or more café-au-lait macules greater than 5mm in prepubertal children and greater than 15mm in postpubertal children. *
2. Two or more neurofibromas of any type or one or more plexiform neurofibromas or large internal neurofibromas. Some patients present with multiple bilateral spinal root neurofibromas and may not meet other criteria; consider mutation testing and/or expert opinion. *
3. Two or more iris Lisch nodules identified by slit lamp examination, or indirect ophthalmoscopy in very young children who cannot cooperate with slit lamp examination. *
4. A heterozygous pathogenic variant in unaffected tissue such as white blood cells.*
5. Bilateral freckles in axilla, groin, neck, sub mammary region in women and peri-oral region. IF diagnosis is based on pigmentary features alone consider genetic testing and/or expert opinion. *
6. Two or more choroidal abnormalities (CAs) - defined as bright, patchy nodules imaged by optical coherence tomography (OCT)/Near-Infrared Reflectance (NIR) imaging.
7. Optic pathway glioma.
8. A distinctive osseous lesion such as: sphenoid wing dysplasia; anterolateral bowing of tibia (tibial dysplasia); or pseudarthrosis of a long bone.

9. A first-degree relative (parent, sibling or child) with NF1 by the above criteria. If only two affected sibs, consider genetic testing and/or expert opinion.

*Patients with somatic mosaicism of the *NF1* gene can present with localized or segmental disease. The asterisks denote criteria that are commonly seen/affected by segmental/mosaic NF1. This includes isolated plexiform neurofibromas +/- local bone changes. If the disease features are unilateral or limited to one or more body segments, consider mosaicism.

RATIONALE – the current criteria have been useful for over 30 years and before major changes we need an evidence base.

9. QUESTION- Do you prefer that some erroneous diagnoses would be avoided by moving to A (major) and B (minor) criteria as below?

To make a diagnosis of NF1 you need two A criteria OR one A and one B. The criteria are:

A criteria:

- Six or more café-au-lait macules greater than 5mm in prepubertal children and greater than 15mm in post-pubertal children. *
- Two or more neurofibromas of any type or one or more plexiform neurofibromas or large internal neurofibromas. Some patients present with multiple bilateral spinal root neurofibromas and may not meet other criteria; consider mutation testing and/or expert opinion. *
- A heterozygous pathogenic variant in unaffected tissue such as white blood cells.*
- A first-degree relative (parent, sibling or child) with NF1 fulfilling diagnostic criteria. If only two affected sibs, consider genetic testing and/or expert opinion.

B criteria:

- Bilateral freckles in axilla, groin, neck, sub mammary region in women and peri-oral region. IF diagnosis is based on pigmentary features alone consider genetic testing and/or expert opinion. *
- Two or more iris Lisch nodules identified by slit lamp examination, or indirect ophthalmoscopy in very young children who cannot cooperate with slit lamp examination. *
- Optic pathway glioma.
- Two or more choroidal abnormalities (CAs) - defined as bright, patchy nodules imaged by optical coherence tomography (OCT)/Near-Infrared Reflectance (NIR) imaging.
- A distinctive osseous lesion such as: sphenoid wing dysplasia; anterolateral bowing of tibia (tibial dysplasia); or pseudarthrosis of a long bone.

*Patients with somatic mosaicism of the *NF1* gene can present with localized or segmental disease. The asterisks denote criteria that are commonly seen/affected by segmental/mosaic NF1. This includes isolated plexiform neurofibromas +/- local bone changes. If the disease features are unilateral or limited to one or more body segments, consider mosaicism.

RATIONALE - The A criteria are those that are either present in over 90% of people with NF1 or are both specific and sensitive. The exceptions are parent fulfilling diagnostic criteria (A criterion) and Lisch nodules which is a B criterion. This is because more common iris lesions may be confused with them. Please comment on this if you disagree. The reason for this model is to avoid erroneous diagnoses of full blow NF1 in people with mosaic disease.

**Supplementary data 4**. NF experts who were involved in at least one step of the revision process.

<u>Clinical Epidemiology</u>

- Nilton Rezende – Federal University of Minas Gerais, Brazil

<u>Clinical Genetics:</u>

- Dusica Babovic-Vuksanovic – Mayo Clinic, USA
- Shay Ben-Shachar – Clalit Research Institute, & Schneider Children's Medical Center, Ramat-Gan, Israel
- Yemima Berman – Royal North Shore Hospital, University of Sydney, Australia
- Ignacio Blanco – Hospital Universitari Germans Trias I Pujol, Badalona, Spain
- Maurizio Clementi – University of Padova, Italy
- D. Gareth Evans – University of Manchester, UK
- Jan Friedman – University of British Columbia, Canada
- Dorothy Halliday - Oxford University Hospitals NHS Foundation Trust, UK
- Arvid Heiberg – Oslo University Hospital, Norway
- Susan M. Huson – Manchester, UK
- Bruce Korf – University of Alabama at Birmingham, USA
- Eric Legius –University Hospitals Leuven, Belgium
- Joanne Ngeow – Lee Kong Chian School of medicine, Nanyang Technological University, Singapore and Cancer Genetics Service, National Cancer Center, Singapore
- Katherine Rauen – University of California Davis, USA
- Vincent Riccardi –The Neurofibromatosis Institute, USA
- Elizabeth Schorry – Cincinnati Children’s Hospital Medical Center, USA
- David Stevenson – Stanford University, USA
- Eva Trevisson – University of Padova, Italy

- David Viskochil – University of Utah, USA

Dermatology:

- Sebastien Barbarot – Centre Hospitalier Universitaire de Nantes, France
- Didier Bessis – Centre Hospitalier Universitaire de Montpellier, France
- Margarita Larralde – Hospital Aleman, Argentina
- Lu Le – University of Texas, Southwestern, USA
- Sirkku Peltonen – University of Turku, Finland
- Dominique Pichard – National Institutes of Health/National Institute of Arthritis and Musculoskeletal and Skin Diseases, USA
- Gianluca Tadini – University of Milan, Italy
- Pierre Wolkenstein – Hôpital Henri-Mondor, France

Genetic Counselor:

- Amanda Bergner – Columbia University Medical Center, USA
- Alicia Gomes – University of Alabama at Birmingham, USA
- Amy Mueller – Massachusetts General Hospital, USA
- Amy Taylor – Cambridge University Hospitals NHS Foundation Trust, UK

Health Services Research:

- Vanessa L. Merker – Massachusetts General Hospital, USA

Internal Medicine:

- Luiz Oswaldo Carneiro Rodrigues – Federal University of Minas Gerais, Brazil

Scientists:

- Marco Giovannini – University of California Los Angeles, USA
- Juha Peltonen – University of Turku, Finland

- 

Eduard Serra - The Institute for Health Science Research Germans Trias i Pujol (IGTP), Spain

Molecular Genetics:

- Hildegard Kehrer-Sawatzki – University of Ulm, Austria
- Conxi Lazaro - Institut Català d'Oncologia (ICO-IDIBELL-CIBERONC), Hospitalet de Llobregat, Spain
- Ludwine Messiaen – University of Alabama at Birmingham, USA
- Laura Papi – University of Florence, Italy
- Meena Upadhyaya – Cardiff University, Wales, UK
- Katharina Wimmer – Institute of Human Genetics, Medical University of Innsbruck, Austria
- Miriam J. Smith – University of Manchester, UK

Neurology:

- David H. Gutmann – Washington University, USA
- Rosalie Ferner – Guy's and St. Thomas' NHS Foundation Trust, UK
- Oliver Hanemann – Peninsula Medical School, University of Plymouth, UK
- Victor Mautner – University of Hamburg – Eppendorf, Germany
- Allyson Parry – John Radcliff Hospital, UK

Neuro-Oncology:

- Jaishri Blakeley – Johns Hopkins University Hospital, USA
- Justin T. Jordan – Massachusetts General Hospital, USA
- Scott Plotkin – Massachusetts General Hospital, USA

Neurosurgery:

- 

- Patricia Ciavarelli – Hospital de Clinicas Gral San Martin, Argentina
- Michel Kalamarides – Hopital Pitie Salpetriere, France
- Michael Link – Mayo Clinic, USA
- Hao Wu - Shanghai Ninth People's Hospital Affiliated Shanghai Jiao Tong University School of Medicine, China
- Gelareh Zadeh – Toronto Western Hospital, Princess Margaret Cancer Centre, Canada

Ophthalmology:

- Robert A. Avery – Children's Hospital of Philadelphia, USA
- Catherine Cassiman – University Hospitals Leuven, Belgium

Pathology:

- Karin Soares Cunha – Universidade Federal Fluminense, Brazil
- Anat Stemmer-Rachamimov – Massachusetts General Hospital, USA

Pediatrics:

- Robert Listernick –Ann and Robert H Lurie Children's Hospital of Chicago, USA
- Rianne Oostenbrink – Erasmus University Medical Center, Netherlands

Pediatric Hematology and Oncology:

- Michael J. Fisher – The Children's Hospital of Philadelphia, USA
- Matthias Karajannis – Memorial Sloan Kettering Cancer Center, USA
- Christopher Moertel – University of Minnesota, USA
- Ali Varan – Hacettepe University,Turkey

Pediatric Neurology:

- Mia MacCollin – Bend, Oregon, USA

- 

- Roger Packer – Children's National Medical Center, USA
- Tena Rosser – Children's Hospital Los Angeles, USA
- Martino Ruggieri - Unit of rare Diseases of the Nervous System, Department of Clinical and Experimental Medicine, Section of Pediatrics and Child Neuropsychiatry, University of Catania, Italy
- Nicole Ullrich – Boston Children's Hospital, USA
- Kaleb Yohay - NYU Langone Health, USA

Pediatric Neuroradiology:

- Stavros Michael Stivaros – University of Manchester, UK
- James Tonsgard – University of Chicago

**Supplementary data 5**. Scoring of revised criteria by non-NF specialists

The statements were rated on a scale from 1 (strongly disagree) to 5 (strongly agree). The specialties of participants included neurology, dermatology, clinical genetics, human genetics, pediatrics.

Q1: The overall rationale for revising the diagnostic criteria for NF1, NF2, and Schwannomatosis are compelling.

- Median = 5 (strongly agree)
- Comment 1: "Molecular data are now available which give additional benefits over clinical criteria alone: make earlier diagnosis possible (NF1) plus give diagnostic clarity for confusing clinical presentations (NF2-Schwannomatosis syndromes or NF1-Legius)."
- Comment 2: "Well explained with clear reasoning and backed up with excellent evidence."

Q2: The recommended changes to the existing diagnostic criteria for NF1 are reasonable.

- Median = 5 (strongly agree)
- Comment 1: "With reservation regarding the requirement for bilateral axillary freckling before this can be counted as a diagnostic criterion. If have bilaterally distributed CAL, the presence of unilateral axillary or inguinal freckling alone is still far more likely to be

non-mosaic than mosaic NF1. Delineating the criteria for mosaic NF1 v non-mosaic NF1 requires clinical expertise in NF1 that guidelines are not easily adapted to. I would prefer a caveat such as "where there is only unilateral freckling with CALM mainly limited to one contiguous region, consider mosaic NF1".

- Comment 2: “Very helpful when assessing young children.”

Q3: I expect non-specialists (i.e. clinicians without a focus on NF) will be able to use the diagnostic criteria for NF1 without difficulty.

- Median = 4.0 (agree)
- Comment 1: “With exception of deciding re mosaic NF1 as before. ”
- Comment 2: “I suspect that most will continue to refer for a more definitive dx.”
- Comment 3: “I think non-specialists sometimes have difficulty in recognizing a CAL patch. Would it be helpful to include some "typical" appearances as photos, or give a more general description.”

Additional comments: NF1

- There is an apparent paradox in the paragraph focused on the term “freckling”. On one hand, the reader understands that it is important to consider genetic mosaicism if the freckling remains unilateral and, on the other hand, in the revised criteria the criterion was adapted to bilateral freckling. This should be clarified.

- In the era of medical genomics, especially for diseases characterized by a wide phenotypic variability, it is questionable to use, at the same level, clinical and molecular criteria. It might be more appropriate to dichotomize them into clinical and molecular criteria.
- It would be important to homogenize the molecular genetics wording and presentation through the text. In contrast to schwannoma predisposition syndromes, it is not specified for NF1 in which tissue the pathogenic variant should be detected. Therefore, I would suggest “NF1 pathogenic variant in an unaffected tissue, such as blood”.
